# Supplementary material for: Predicting visual function by interpreting a neuronal wiring diagram
Source: Nature. 2024 Oct 2;634(8032):113–23. doi: 10.1038/s41586-024-07953-5 (PMC11446822; doi:10.1038/s41586-024-07953-5)
Supplement: Supplementary file 6 — CRF and ERF predictions for individual TmY4 and TmY9 cells. Analogous to Supplementary Data 3, but for TmY target types. Shown are the top four monosynaptic pathways, the strongest pathway passing through each of the top ten intermediary types (ranking from Extended Data Fig. 7), and the trisynaptic pathway Tm1–TmY–Dm3–TmY (see the section entitled Prediction of spatial normalization). [file 41586_2024_7953_MOESM6_ESM.zip › DataS4/TmY9q/720575940615991820.pdf]

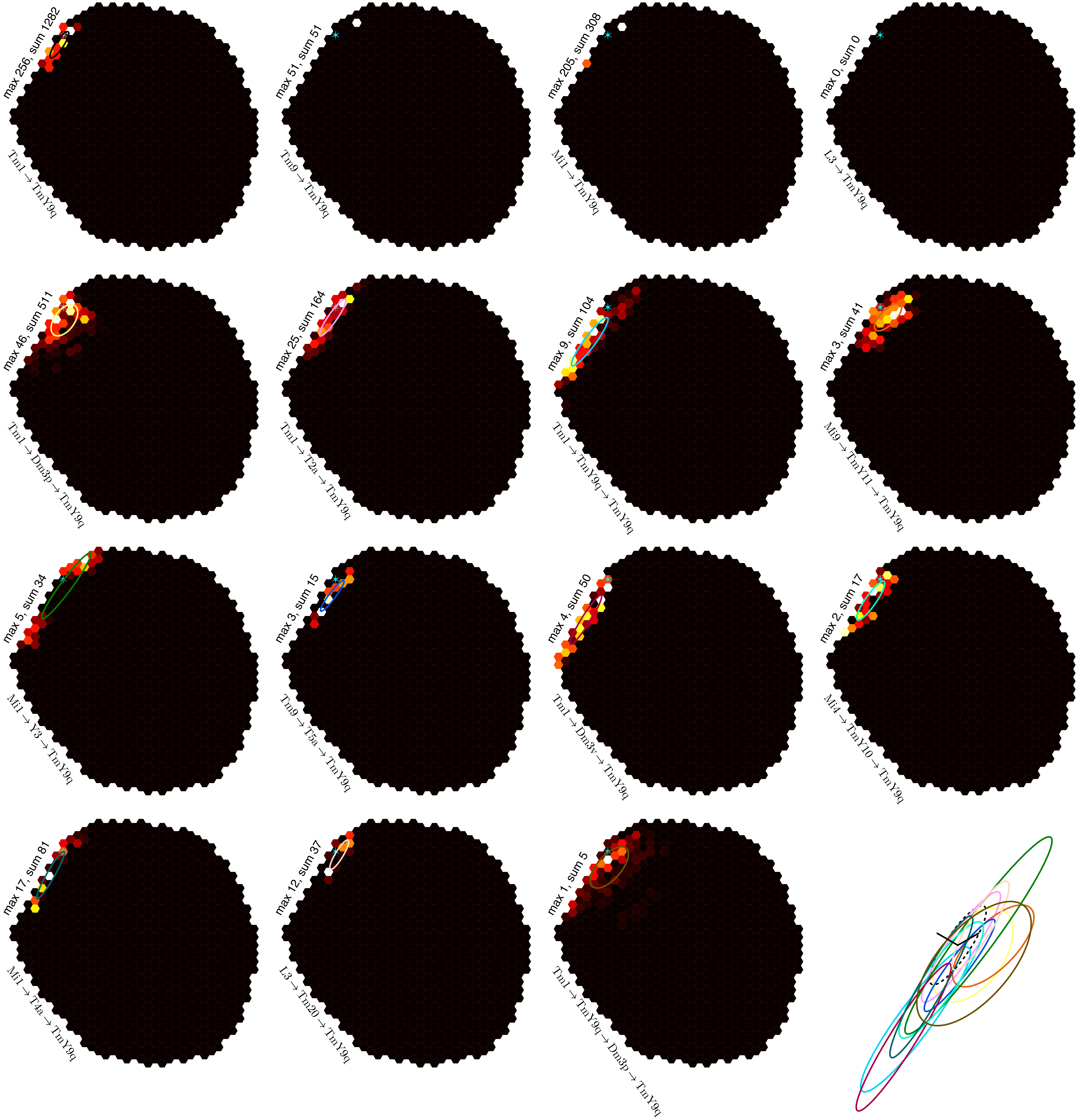
max 256, sum 1282
Tm1 → TmY9q
max 51, sum 51
Tm9 → TmY9q
max 205, sum 308
Mi1 → TmY9q
max 0, sum 0
L3 → TmY9q
max 46, sum 511
Tm1 → Dm3p → TmY9q
max 25, sum 164
Tm1 → T2a → TmY9q
max 9, sum 104
Tm1 → TmY9q → TmY9q
max 3, sum 41
Mi9 → TmY11 → TmY9q
max 5, sum 34
Mi1 → Y3 → TmY9q
max 3, sum 15
Tm9 → T5a → TmY9q
max 4, sum 50
Tm1 → Dm3v → TmY9q
max 2, sum 17
Mi4 → TmY10 → TmY9q
max 17, sum 81
Mi1 → T4a → TmY9q
max 12, sum 37
L3 → Tm20 → TmY9q
max 1, sum 5
Tm1 → TmY9q → Dm3p → TmY9q
